# Toxicity and genotoxicity in *Astyanax bimaculatus* (Characidae) induced by microcystins from a bloom of *Microcystis* spp

**DOI:** 10.1590/s1415-47572010000400023

**Published:** 2010-12-01

**Authors:** Ricardo Rocha Pavan da Silva, Osmindo Rodrigues Pires, Cesar Koppe Grisolia

**Affiliations:** 1 Departamento de Genética e Morfologia, Instituto de Ciências Biológicas, Universidade de Brasília, Brasília, DFBrazil; 2 Departamento de Ciências Fisiológicas, Instituto de Ciências Biológicas, Universidade de Brasília, Brasília, DFBrazil

**Keywords:** microcystin, comet assay, fish micronucleus, necrosis, apoptosis

## Abstract

Studies of genotoxicity in fish caused by cyanobacterial microcystins can be useful both in determining the sensitivity of native species, as well as comparing exposure routes. The genotoxicity caused by the microcystins LR and LA from a bloom collected in a eutrophic lake, was revealed in the fish *Astyanaxbimaculatus*, a native species from South America. LC50 (72 h) was determined as 242.81 μg L ^-1^ and LD50 (72 h) as 49.19 μg kg ^-1^ bw. There was a significant increase of DNA damage in peripheral erythrocytes, following intraperitoneal injection (ip) with tested concentrations of 24.58 μg kg ^-1^ bw and 36.88 μg kg ^-1^ bw, as well as through body exposure to a concentration of 103.72 μg L ^-1^ . Micronucleus (MN) induction was observed after ip injections of 24.58 μg kg ^-1^ bw and 36.88 μg kg ^-1^ bw for 72 h, as well as following body exposure for 72 at 103.72 μg L ^-1^ . Thus, both exposure routes resulted in MN induction and DNA damage. Apoptosis-necrosis testing was carried out only by ip injection with concentrations of 24.58 μg kg ^-1^ bw and 36.88 μg kg- 1 bw. Exposure to microcystins at lower concentrations induced more apoptosis than necrosis in peripheral erythrocytes, whereas exposure at higher concentrations gave rise to both conditions. Thus, *Astyanax bimaculatus* can be considered as a species sensitive to the genotoxic effects caused by microcystins.

## Introduction

Cyanotoxins are a diverse group of natural toxins produced by cyanobacteria which can be found in lakes, ponds and rivers. They are hepatotoxic, neurotoxic and dermatotoxic, with effects on the inhibition of protein synthesis causing serious ecological and human health problems. Moreover, they are inhibitors of PP1 and PP2 phosphatases, especially in the liver, thereby causing morphological damage, starting with cytoskeletal disruption and loss of sinusoidal structure. Liver weight increases due to intrahepatic hemorrhage, followed by hemodynamic shock, heart failure, and finally death by hemorrhagic shock (Eriksson *et al.*, 1990; Chorus and Bartram, 1999).

Erythrocyte cells from peripheral blood are commonly used for the application of the comet assay in conjunction with micronucleus testing, in fish species exposed to water pollutants (Russo *et al.*, 2004). *Astyanax bimaculatus*, a nonmigratory native fish species, commonly known as the ‘lambari', is widely abundant throughout South America. The species, besides playing a central role in riverine food webs, is sensitive to environmental degradation. These characteristics make it an excellent bio-indicator, through being both commonly present in Brazilian ponds and rivers, as well as a potential target of microcystins from cyanobacterial bloom.

Lake Paranoá, a tropical reservoir, was built in 1959, simultaneous with the construction of the Brazilian capital, Brasilia. Within a decade, the lake became eutrophic due to inadequate sewage treatment associated with high population growth (Altafin *et al.*, 1995). Nutrient input from domestic sewage is still the main source of pollution in Brasília, a city without any chemical industry. The lake is typically eutrophic, with phosphorus representing the limiting nutrient for algal growth (CAESB, 1996). The presence of the cyanotoxin producer *Microcystis aeruginosa* was revealed through a water-monitoring program undertaken by the Municipal Drinking Water and Sewage Corporation (CAESB).

The bioaccumulation of microcystins in fish was first observed in salmon after eating crab larvae containing microcystin (Williams *et al.*, 1997) The accumulation in the liver and muscles of *T. rendalli* was demonstrated by Soares *et al.*, (2004), who also showed that these toxins could still be found in the muscles of the fish several days after contamination. *Astyanax bimaculatus*, besides its use as fishing bait, is also a snack-food, an ornamental fish and a larva-eater in combating mosquito larvae. Furthermore, through being herbivorous and phytoplanktivorous, it is incapable of avoiding the ingestion of toxic compounds. The aim hereby was to evaluate the toxicity and genotoxicity to *Astyanax bimaculatus*, as induced by an extract of cyanobacterial microcystins, using two administration routes and different endpoints, such as micronucleus and apoptosis-necrosis testing, and comet assaying.

## Material and Methods

The specimens of *Astyanax bimaculatus* used in this study were obtained from a local fish farm, where breeding and sanitary conditions were under constant control and monitoring. The criterion for fish-selection was a body-length of 7-10 cm. The fishes were acclimatized for a week in 250 L tanks in the Genetics Laboratory of the University of Brasilia, with continuously aerated, filtered and dechlorinated tapwater. They were kept at constant temperature (25 ± 2 °C), conductivity (550 ± 50 μS), pH (7.0 ± 0.5) and photoperiod (14:10 light:dark), with twice-a-day feeding with granular fish-chow. The level of ammonium in the water was constantly monitored and the water itself periodically renewed. The fishes, in groups of eight, were randomly placed in glass aquaria of 30 L. Treatments consisted of intraperitoneal (ip) injection and body exposure. To determine the toxicity (LC50-72 h and LD - 72 h) the Trimed Spearman-Karber method was used (Hamilton *et al.*, 1977). Treatments with the extract were undertaken with the following concentrations: 54.2 μg kg^-1^ bw, 36.88 μg kg^-1^ bw, and 24.58 μg kg^-1^ bw for 72 h in the ip injection, and 103.72 μg L^-1^ and 414.90 μg L^-1^ for 72 h in body-exposure, plus the respective control in each case. Micronucleus tests, comet assays and necrosis versus apoptosis tests were carried out with peripheral-blood erythrocytes. The research project was approved by the Animal Ethical Committee of the University of Brasilia.

###  Characterization of the extract

**Figure 1:**
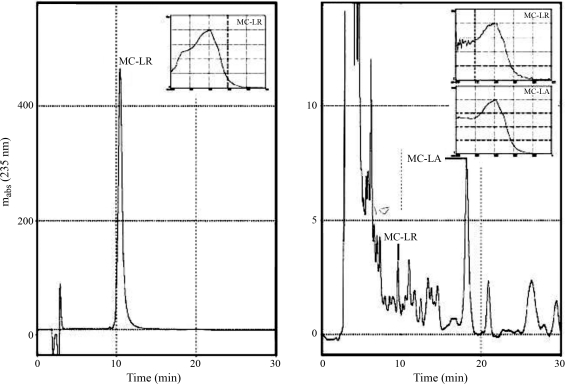
Chromatogram with standard microcystin-LR (left) and the studied extract from a bloom of cyanobacteria showing the strong presence of microcystin-LR and at second concetration level microcystin-LA (right)

The extract was obtained from bloom collected in Lake Paranoá on June 25, 2006. The flowering material was lyophilized and then sonicated, whereby the cyanobacterium cells were fractured, with the subsequent elimination of toxins. Thereafter, a small aliquot was removed from the sample for characterization by HPLC. The identification of toxins produced by the bloom of *Microcystis* spp was by comparing the chromatographic fraction for standard microcystin-LR (SIGMA, CO), with the retention time in the chromatography system and index of similarity of the spectrogram in the range of microcystin absorbance of 200 to 300 nm (Figure 1). Fractions for the two variants of microcystin produced by the bloom of *Microcystis* spp and identified by HPLC-PDA system analysis were fragmented by mass spectrometry – MALDI-TOF/TOF.

A calibration curve for microcystin was prepared from values obtained through chromatographic analysis. The presence of -LR and -LA microcystins was detected by the HPLC system in 300 mL of *Microcystis* spp bloom extract. Based on this, the standard curve was calculated from a concentration of 29.19 mg of microcystin-LA and 12.30 mg of microcystin-LR. There was, therefore, a total of 138.3 g mL^-1^ of microcystins in the tested extract. It is noteworthy that other substances can occur in the extract, since the methodology presented is able to detect only the microcystins but no other type of chemical compound.

###  Micronucleus testing

Peripheral blood (50 μL), obtained by cardiac puncture with a heparinized syringe, was immediately smeared. After fixation in ethanol for 15 min, slides were left to air-dry, whereupon they were stained with acridine orange at a concentration of 0.003%. The stained slides were viewed under an epi-fluorescent microscope at 1000X magnification, and checked for the presence of micronuclei exhibiting yellow-green fluorescence in the peripheral blood erythrocytes. For each treatment, all eight fish were sampled and 3,000 erythrocyte cells with complete cytoplasm were scored per fish (24,000 cells per treatment). The criteria for identifying micronucleated erythrocytes were: (a) MN should be one-third smaller than the main nuclei; (b) MN must not touch the main nuclei; (c) MN must be of the same color and intensity as the main nuclei. These data were statistically analyzed by non-parametric Mann-Whitney *U*-test, considering α = 5%.

###  Comet assaying

This was undertaken as described by Singh *et al.* (1988), but with certain modifications. The cell-suspension sampled in the microtubule was mixed with 120 μL of low melting agarose (37 °C). Then, 500 μL of the erythrocyte-agarose suspension were placed onto a fully frosted slide pre-coated with standard agarose (1.5%), and covered with a coverslip. The slides were then kept on ice for 15 min to permit complete agarose polymerization, and afterwards inserted into a chilled lysing solution (NaCl 2.5 M; EDTA 100 mM; Tris 10 mM; N-laurolyl-sarcosine 1%; Triton-X 1%; DMSO 10%; pH 10). Then the slides were then placed onto a horizontal gel electrophoresis platform and covered with a chilled alkaline solution consisting of 300 mM of NaOH and 1 mM of Na_2_EDTA (pH 13); they were left in the dark at 4 °C for 30 min, and then the DNA was electrophoresed at 4 °C in the dark for 30 min at 25 V and approximately 350 mA. The slides were gently rinsed twice with 400 mM Tris (pH 7.5) to neutralize the alkali. Each slide was stained with 30 μL of 20 μg mL^1^ ethidium bromide and covered with a coverslip. One hundred cells from each replicate were randomly chosen (50 from each duplicate slide), and analyzed under an optical fluorescence microscope (Axioskop-2, Carl Zeiss), with a 510-560 nm filter and a 590 nm barrier filter, at a magnification of 400x.

For damage index calculation, cells were sorted into four classes, according to tail size. The index of damage (ID) is the sum of the classes of 100 cells analyzed per fish, and may vary from 0 (all the cells are undamaged - 0X100) to 400 (all the cells are highly damaged - 4X100). The damage index is based on the length of migration and the amount of DNA in the tail, and it is considered a sensitive measurement of detectable DNA damage. Statistical analysis was carried out with the MINITAB program, using the ANOVA parametric test and Tukey parametric linear correlation, with a significance level of 95%. The following formula was used to quantify DNA damage:



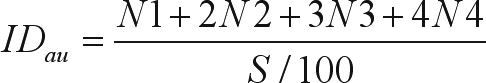


where *ID* is the index of DNA damage, *au* an arbitrary unit, *N*1 - *N*4 the nucleoids in levels 1, 2, 3 and 4, *S* the number of nucleoids analyzed, including level 0.

###  Apoptosis-necrosis testing

Treatments were carried out in groups of eight fishes through the intraperitoneal injection of an extract of *Microcystis* spp at 36.88 μg kg^-1^ bw and 24.58 μg kg^-1^ bw for 72 h. 0.1 mL of peripheral blood was obtained from cardiac puncturing and diluted in 2.0 mL of fetal bovine serum at room temperature (22 ± 2 °C). 15 μL of cell suspension was immediately smeared onto slides, whereupon 1 μL of acridine orange/ethidium bromide (1:1) stain was added and the slides covered with a coverslip. The slides were analyzed with a fluorescence Axioskop 2 Zeiss microscope at 1000X magnification using a wavelength of 510-560 nm. Five hundred peripheral erythrocytes were analyzed and classified as viable, necrotic or apoptotic. Results were statistically analyzed by t-test, with significance of 0.05.

## Results

In the toxicological test of LD 50 with *A. bimaculatus,* 6 out of 10 fishes died after ip application at a concentration of 54.20 μg kg^-1^ bw, thus making this unfeasible for evaluating genotoxicity. Low mortality was observed in the concentrations 24.58 μg kg^-1^ bw and 36.88 μg kg^-1^ bw, with 2 deaths out of 10 fishes exposed in each concentration. LD50 (72 h) was determined as 49.19 μg kg^-1^ at a confidence interval of 38.58 μg kg^-1^ - 62.73 μg kg^-1^. The LC50 (72 h) was determined as 242.81 μg kg^-1^ with a confidence interval of 152.74-386.00 μg L^-1^. In the micronucleus tests, exposure via ip was statistically significant for both tested microcystin concentrations of 24.58 μg kg^-1^ and 36.88 μg kg^-1^ bw. Body exposure experimenting also showed micronucleus induction at a concentration of 103.72 μg L^-1^ (Table 1). Comet assaying also revealed genotoxicity along both routes of exposure (Table 2). Apoptosis-necrosis testing showed that only apoptotic erythrocyte cells were found at lower concentrations, whereas both necrosis and apoptosis were found at the higher ones (Table 3).

## Discussion

Several well-known exotic species of fish are worldwide exposed to mutagens in water and used as bioindicators of genotoxicity, such as *Oreochromis niloticus*, *Cyprinus carpio*, *Daniorerio*, *Carassius auratus*, etc. However, in field situations, pollutants dissolved or suspended in the water require an appropriate native species as *in situ* bioindicator. Much evidence has indicated that not only mammals are susceptible to cyanotoxins, but fish as well (Jos *et al.*, 2005). In cyanobacteria blooms, deaths of fish were observed mainly involving planktivorous species, such as C*yprinus carpio* (Rodger *et al.*, 1994). Fisher and Dietrich (2000) showed the pathological and biochemical characterization of microcystin-induced hepatopancreas and kidney damage in carp (*Cyprinus carpio*).

Andersen *et al.* (1993) reported mortality at 555 pg kg^-1^ in salmon (*Salmo salur*) injected three times via ip with microcystin-LR. In rainbow trout the LD50 of microcystin-LR is between 400 and 1000 mg kg^-1^ (Kotak *et al.*, 1996). In our study, the LC50 and LD50 observed in *A. bimaculatus* were 242.81 mg L^-1^ and 49.19 μg kg^-1^ bw, respectively, thus demonstrating a considerable difference in susceptibility to microcystin-LR in this species. In addition, the fishes' nutritional and physiological conditions can have an affect on cyanotoxin toxicity (Rabergh *et al.*, 1991). Normally, the effect of oral administration of a toxicant is approximately 10 times less than via ip (Carbis *et al.*, 1996). Zhao *et al.* (2006) found a consumption of 1500-6000 μg kg^-1^ bw of microcystin by tilapia to be equivalent to 150-600 μg kg^-1^ bw injected via ip, this without affecting the growth or feeding rate of the species. In a species of carp (*Carassius auratus gibelio*) high mortality occurred when fish were fed on diets containing low doses of microcystin (1.02-10.76 μg kg^-1^ bw) (Zhao *et al.*, 2005).

In MN tests, significant statistical differences were found for both routes of exposure. Only one concentration (103.72 μg L^-1^) was used for body exposure testing, as this was considered to be the maximum tolerated dose when based on the LC50. *A. bimaculatus* proved to be more sensitive to MN induction through ip than through body exposure. Comet assaying has been successfully applied under both laboratory and field conditions, through being a non-specific, sensitive, rapid and economical biomarker in the detection of genetic damage in natural biota (Jha, 2008). This author also inferred that comet assaying is capable of detecting oxidized DNA bases in fishes exposed to environmental contaminants. The metabolism of microcystins in animals gives rise to the formation of reactive oxygen species, such as superoxide anion radicals, hydrogen peroxide and hydroxyl radicals (Ding *et al.*, 2000, 2001). Comet results followed the MN results, also demonstrating a dose-effect relationship in ip exposure. Though intraperitoneal injection is an inappropriate route for fish models in genotoxicity studies, it normally bestows a more precise exposure level to studied toxins, and therefore better response, than through aquatic exposure.

Apoptosis due to DNA oxidative damage by microcystins in animal cells has been extensively reported both *in vivo* and *in vitro* (Gehringer, 2004). Yin *et al.* (2006) showed that microcystins induce oxidative damage in tobacco BY-2 cells exposed *in vitro*. In apoptosis-versus-necrosis assaying, we observed that microcystin exposure at the highest concentration induced a statistically significant level of both necrosis and apoptosis, whereas only apoptosis occurred on exposure at the lowest concentration. Our data showed that a microcystic extract, when in low concentrations, could activate cellular oxidative stress, thereby causing genotoxicity but not toxicity, as proposed by several of the above cited authors. Obviously, cell-death by necrosis occurred due to exposure to a high concentration of a well-known toxic compound. Therefore, cyanobacterial bloom in ponds represents a genotoxic risk to fish, and consequently to human health, due to bioaccumulation.

## Figures and Tables

**Table 1:** Means (SD) of micronuclei in *Astyanax bimaculatus* after treatment with extracts at different concentrations, considering two exposure routes.

Treatments	Exposure route	MN (Mean ± SD)	P Mann Whitney-U test
Control		0.6 ± 0.7	
103.72 μg/L	Body exposure	2.9 ± 1.85	0.002 *
24.58 μg kg^-1^ bw	i.p.	2.5 ± 1.41	0.008 *
36.88 μg kg^-1^ bw	i.p.	3.5 ± 2.62	0.024 *

*Significant at level of 5%.

**Table 2:** Means (SD) of DNA damage index obtained from comet assay from peripheral erythrocytes of *Astyanax bimaculatus* after treatment with extracts with different concentration, considering two exposure routes.

Treatments	Exposure route	DNA damage (Mean ± SD)	P Mann Whitney-U test
Control		30.57 ± 16.03	
103.72 μg/L	Body exposure	205.30 ± 38.41	0.0004 *
24.58 μg kg^-1^ bw	i.p.	70.13 ± 29.49	0.0046 *
36.88 μg kg^-1^ bw	i.p.	122.27 ± 26.16	0.0012 *

*Significant at level of 5%.

**Table 3:** Means (SD) of viables, necrotic and apoptotic peripheral erythrocyte cells of *Astyanax bimaculatus* and their respective percentages after ip injection of extracts of *Microcystis* spp.

Treatments	Viable	Necrosis	Apoptosis	% Viability	% Necrosis	% Apoptosis
Control	473.9 ± 29.3	20.9 ± 28.9	5.3 ± 3.6	94.8	4.2	1.1
24.58 μg kg^-1^ bw	266.1 ± 155	18.4 ± 15.6	16.6 ± 9.9	87.4	6.0	5.5*
36.88 μg kg^-1^ bw	79.1 ± 10	15.6 ± 9.1	5.4 ± 1.6	79.1	15.6*	5.4*

*Significant at level of 5%.
